# A cross-sectional study on clinical vigilance in the diagnosis and treatment of listeriosis among pregnant women and their knowledge, attitudes, and practices regarding listeriosis in Gansu Province, China

**DOI:** 10.3389/fpubh.2025.1661344

**Published:** 2026-01-08

**Authors:** Wen-Xuan Lin, Kai-Li Wang, Xiang-Lai Sang, Zhen-Yin Shi, Xiao-Cheng Liang

**Affiliations:** Department of Nutrition and Food Safety, Gansu Provincial Center for Disease Control and Prevention, Lanzhou, China

**Keywords:** listeriosis, physicians, diagnosis and treatment, pregnant women, knowledge-attitude-practice survey

## Abstract

**Background:**

Listeriosis is a serious foodborne disease that threatens the health of pregnant women and their fetuses. Gansu Province, in northwest China, is economically underdeveloped and covers a large geographic area. No population-based studies on listeriosis have been conducted there. In 2022, Gansu added listeriosis to its foodborne disease surveillance system and started a pilot program at five tertiary hospitals in four cities. By the end of 2024, 13 confirmed cases had been reported, including three linked to pregnancy: one miscarriage and two preterm births at 27 weeks and 34 weeks plus 2 days, respectively.

**Objectives:**

This study aims to assess the clinical vigilance for listeriosis in Gansu Province and to investigate pregnant women’s knowledge, attitudes, and practices (KAP) regarding the disease.

**Methods:**

Eight tertiary hospitals were selected as research sites. A convenience sampling method was used to survey 207 physicians from obstetrics, emergency medicine, and gastroenterology departments, along with 589 pregnant women receiving prenatal care. Descriptive statistics were generated using WPS Office 10.8.0, while SPSS 21.0 was employed for ANOVA, Spearman correlation analysis, and multiple linear regression modeling.

**Results:**

The proportion of physicians who had treated listeriosis patients and those who had participated in relevant training was identical at 14.98%. Failure to diagnose the disease was identified as the primary reason for underreporting among clinicians. A low percentage of physicians were aware of foods commonly contaminated by *Listeria monocytogenes*, as well as the main clinical symptoms and recommended treatment options for listeriosis. Over half of the surveyed pregnant women reported cleaning their refrigerators no more than twice per year, consumed high-risk foods within 4 weeks prior to the survey, and failed to separate raw and cooked foods on cutting boards at home. Pregnant women with lower-educated pregnant women showed higher rates of using the same cutting board for raw and cooked foods and poor handwashing habits. All differences were statistically significant (χ^2^ = 13.177, 9.939; all *p* < 0.05), Those first-time pregnant women were more likely to eat at mobile food stalls, consume high-risk foods, and clean refrigerators≤2 times/year than non-first-time mothers (χ^2^ = 4.267, 10.436, 14.150; all *p* < 0.05). Higher overall scores on listeriosis-related knowledge, attitudes, and behaviors were associated with advanced maternal age, higher education level, increased family income, later gestational stage, and being a first-time mother.

**Conclusion:**

Clinical vigilance for listeriosis diagnosis and treatment among physicians in tertiary hospitals remains limited. Pregnant women exhibit low awareness of listeriosis and engage in high-risk behaviors at elevated rates.

## 1 Introduction

*Listeria monocytogenes* (LM), the main causative agent of human listeriosis, a highly pathogenic intracellular bacterium that demonstrates the characteristics of psychrotrophic growth and flagellum-mediated motility (1). Individuals with compromised immune systems are particularly susceptible to infection (2). Elevated progesterone levels and reduced cellular immunity increase the risk of invasive LM infection during pregnancy. Pregnant women have a 10–20 times higher risk of contracting listeriosis from consuming LM-contaminated food compared to the general population (3), and more than 100 times higher risk than non-pregnant women of reproductive age (4). According to the World Health Organization (WHO), 43% of all reported listeriosis cases occur during pregnancy, with 14% occurring in the third trimester (5). LM can cross the placental barrier, leading to bacteremia in pregnant women and potentially causing severe adverse pregnancy outcomes such as miscarriage, stillbirth, and neonatal infection. From 1964 to 2010, 46 pregnancy-related listeriosis cases were documented in literature in China, while from 2010 to 2023, 482 cases were reported, with neonatal mortality rates of 46 and 27.1%, respectively (6). Despite being a notifiable disease in countries like the United States, Canada, and some EU nations, listeriosis remains non-notifiable in China. Notably, an estimated 99% of listeriosis cases in China are transmitted through food (7). The clinical presentation of listeriosis lacks specificity (8), and blood cultures are positive in only 36% of symptomatic pregnant patients (9), making diagnosis challenging and increasing the likelihood of missed or misdiagnosed cases (10, 11). Therefore, enhancing clinicians’ vigilance of listeriosis, initiating early antimicrobial coverage for LM, and promoting timely detection and treatment are crucial for preventing adverse pregnancy outcomes. Additionally, health education targeting pregnant women plays a vital role in disease prevention. The investigation aims to assess the clinical vigilance for listeriosis and evaluate pregnant women’s knowledge, attitudes, and practices (KAP) in Gansu Province, with the ultimate goal of informing and enhancing listeriosis surveillance and public prevention education. The study integrates on-site survey administration with targeted health education delivered to pregnant women after completing the questionnaire.

## 2 Materials and methods

### 2.1 Participants

Eight tertiary medical institutions were randomly selected across Gansu Province, located in Lanzhou, Qingyang, Wuwei, and Dingxi cities, including three listeriosis monitoring sentinel hospitals. The study population included doctors from the departments of obstetrics, emergency medicine, and gastroenterology, as well as pregnant women receiving prenatal care between September and December 2023.

### 2.2 Sample size determination

Physicians: 220 actively practicing physicians from the departments of obstetrics, emergency medicine, and gastroenterology who provided informed consent. Pregnant women cohort: the sample size was calculated based on an assumed listeriosis vigilance rate of 7.78%, derived from reported prevalence data in Beijing, with a z-score (μα/_2_) of 1.96, a margin of error (*δ*) of 0.05, and a design effect (deff) of 1.5. The minimum required sample size was calculated to be 578, ultimately, 589 participants were enrolled to ensure sufficient statistical power.

### 2.3 Survey instruments

*Physician assessment questionnaire:* Demographic and professional characteristics (institution, department, educational attainment, professional rank); Clinical experience with listeriosis; Knowledge assessment parameters; Therapeutic approaches; Professional training history; Diagnostic challenges and barriers.

*Pregnancy KAP (Knowledge, Attitudes, Practices) questionnaire:* section 1: Demographic profile (age, occupation, education level, household income, parity, gestational age); section 2: Knowledge evaluation (6 items, dichotomous scoring, maximum six points); section 3: Attitude assessment (four items, 3-point Likert scale: positive = 2, neutral = 1, negative = 0, maximum eight points); section 4: Behavioral practices (nine items, binary scoring: safe practice = 1, high-risk behavior = 0, maximum nine points). Note: Multiple-response items were scored as incorrect (0) for any wrong selection, and correct (1) for complete or partially correct responses.

The survey was implemented utilizing convenience sampling with face-to-face questionnaire administration.

### 2.4 Quality assurance protocol

The principal investigator supervised all field operations. Standardized training was delivered to survey staff at all study sites prior to data collection. Quality control measures were implemented as follows:

1. Pre-analysis questionnaire screening for completeness and logical consistency.
2. Double data entry using Epidata software with consistency verification.
3. Random audit of 10% of the questionnaires to ensure data accuracy.

### 2.5 Statistical analysis

Data were exported from Epidata and analyzed using SPSS 21.0. Normally distributed continuous variables were expressed as mean ± standard deviation (x ± s), while non-normally distributed measurement data were presented as median and interquartile range [M (P_25_–P_75_)]. Categorical variables were summarized as frequencies and percentages (%). Group comparisons of categorical variables were conducted using the chi-square test, with Yates’ continuity correction applied where appropriate. Spearman’s rank correlation coefficient was used to assess the correlation between non-parametric continuous variables. A multiple linear regression model was performed to explore the factors influencing pregnant women’s knowledge, attitudes, and practices regarding listeriosis, and corresponding regression coefficients were estimated. Variables were included in the model based on an entry criterion of *α* < 0.05. Statistical significance was defined as a two-tailed *p* value less than 0.05.

## 3 Results

### 3.1 Results of physicians

#### 3.1.1 Characteristics

**Figure 1:**
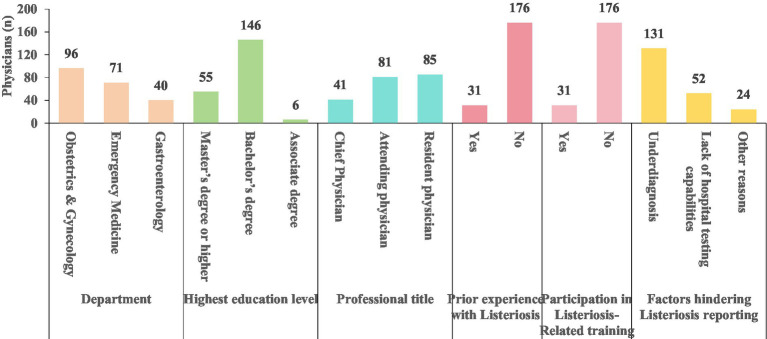
Demographic characteristics of physicians (n).

A total of 220 physicians were surveyed on-site, with 207 valid questionnaires collected, resulting in a recovery rate of 94.1%. The majority of the participants held a bachelor’s degree, representing 70.53% (146/207). The professional titles of “Resident physician” and “Attending physician” accounted for 41.06% (85/207) and 39.13% (81/207), respectively. Among the respondents, 14.98% (31/207) had clinical experience treating patients with Listeria (Physicians with prior experience managing listeriosis cases includes those who encountered such patients not only at their current institution but also during training/rotations at other hospitals), and an equal proportion (14.98%, 31/207) reported having participated in relevant training programs. Failure to diagnose listeriosis was identified as the primary reason for underreporting (The failure to diagnose represents a subjective judgment made by the investigated physician, indicating that the physician perceived a lack of vigilanceregarding the disease), cited by 63.29% (131/207) of the respondents. Additionally, 85.02% (176/207) of the physicians indicated that they had not attended any listeriosis-related training or academic conferences within the past 2 years (Figure 1 and Table 1 for detailed information).

**Table 1:** Demographic characteristics of physicians n (%).

Characteristics	Groups	n (%)
Department	Obstetrics and gynecology	96(46.38)
	Emergency medicine	71(34.30)
	Gastroenterology	40(19.32)
Highest education level	Master’s degree or higher	55(26.57)
	Bachelor’s degree	146(70.53)
	Associate degree	6(2.90)
Professional title	Chief physician	41(19.81)
	Associate chief physician	28(13.53)
	Attending physician	81(39.13)
	Resident physician	85(41.06)
Prior experience with listeriosis cases	Yes	31(14.98)
	No	176(85.02)
Participation in listeriosis-related training	Yes	31(14.98)
	No	176(85.02)
Factors hindering listeriosis reporting	Underdiagnosis	131(63.29)
	Lack of hospital testing capabilities	52(25.12)
	Other reasons	24(11.59)

#### 3.1.2 Physicians’ knowledge and treatment practices regarding listeriosis in pregnant women

**Figure 2:**
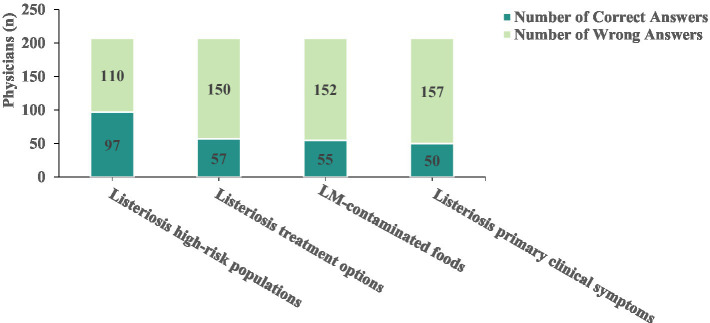
Listeriosis knowledge assessment among surveyed physicians (n).

Among the 207 physicians surveyed on-site, 46.86% (97/207) were aware of the high-risk populations for listeriosis, 27.54% (57/207) had knowledge of the treatment options for listeriosis, 26.57% (55/207) were aware of food items commonly associated with LM contamination, and 24.15% (50/207) recognized the primary clinical symptoms of listeriosis in patients (Figure 2 and Table 2 for further details).

**Table 2:** Listeriosis knowledge assessment among surveyed physicians n (%).

Assessment item	Number of correct answers	Knowledge rate (%)
Listeriosis high-risk populations	97	46.86
Listeriosis treatment options	57	27.54
LM-contaminated foods	55	26.57
Listeriosis primary clinical symptoms	50	24.15

### 3.2 Results of pregnant women

#### 3.2.1 Characteristics

**Figure 3:**
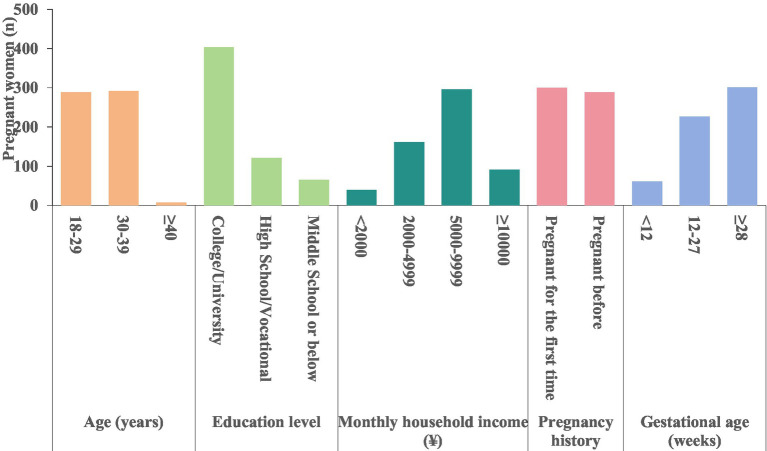
Pregnant participants demographics (n).

This study conducted on-site investigations among 663 pregnant women and collected 589 valid questionnaires, achieving a questionnaire recovery rate of 88.84%. The age range of the participants was 18–49 years, with 49.58% (292/589) falling within the 30–39 age group. Participants with postgraduate, undergraduate, or junior college degrees made up the largest group, accounting for 68.42% (403/589). Approximately half of the participants (50.25%, 296/589) reported a combined monthly household income between 5,000 and 10,000 Chinese Yuan. The ratio of first-time pregnancies to previous pregnancies was 1.04:1. Additionally, 51.10% (301/589) of the respondents were in their third trimester of pregnancy (Figure 3 and Table 3 for detailed information).

**Table 3:** Pregnant participants demographics n (%).

Characteristics	Groups	n (%)
Age (years)	18–29	289(49.07)
	30–39	292(49.58)
	≥40	8(1.36)
Education level	College/university	403(68.42)
	High school/vocational	121(20.54)
	Middle school or below	65(11.04)
Monthly household income (¥)	<2,000	40(6.79)
	2,000–4,999	162(27.50)
	5,000–9,999	296(50.25)
	≥10,000	91(15.45)
Pregnancy history	Pregnant for the first time	300(50.93)
	Pregnant before	289(49.07)
Gestational age (weeks)	<12	61(10.36)
	12–27	227(38.54)
	≥28	301(51.10)

#### 3.2.2 Awareness of listeriosis among pregnant women

**Figure 4:**
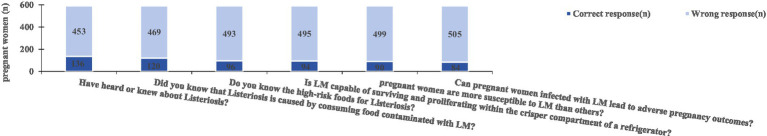
Knowledge of listeriosis among pregnant women.

Among the pregnant women surveyed, only 23.09% (136/589) had heard of or possessed some knowledge about the disease. Of the respondents, 20.37% were aware that listeriosis is caused by consuming food contaminated with LM. A smaller proportion (16.30%) correctly identified high-risk food items, including cooked meat products, Chinese-style cold dishes, raw fruits and vegetables, and Machine-made ice cream. Additionally, 15.96% understood that LM can survive and proliferate in refrigerated environments. Only 15.28% recognized that pregnant women are at higher risk for LM infection compared to the general population. Furthermore, 14.26% were aware that LM infection during pregnancy may result in serious complications such as miscarriage, preterm delivery, stillbirth, or neonatal meningitis. Detailed data are presented in Table 4 and illustrated in Figure 4.

**Table 4:** Knowledge of listeriosis among pregnant women n (%).

Knowledge item	Correct response(n)	%
Have heard or knew about listeriosis?	136	23.09
Did you know that listeriosis is caused by consuming food contaminated with LM?	120	20.37
Do you know the high-risk foods for listeriosis?	96	16.30
Is LM capable of surviving and proliferating within the crisper compartment of a refrigerator?	94	15.96
pregnant women are more susceptible to LM than others?	90	15.28
Can pregnant women infected with LM lead to adverse pregnancy outcomes?	84	14.26

#### 3.2.3 Maternal attitudes toward listeriosis prevention

**Figure 5:**
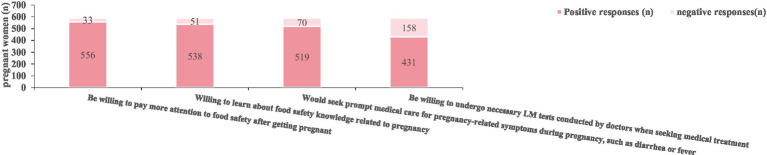
Pregnancy attitudes regarding listeriosis prevention.

Pregnant women demonstrate a generally positive attitude toward listeriosis prevention. Specifically, 94.40% of the participants expressed willingness to pay increased attention to food safety after getting pregnant, and 91.34% indicated readiness to acquire knowledge regarding food safety during pregnancy. Additionally, 88.12% reported they would seek medical care promptly if symptoms such as diarrhea or fever occurred during pregnancy. However, only 73.17% were willing to undergo LM testing when visiting healthcare providers, indicating a relatively lower acceptance rate for this preventive measure (Figure 5 and Table 5).

**Table 5:** Pregnancy attitudes regarding listeriosis prevention.

Preventive behaviors	Positive responses (n)	%
Be willing to pay more attention to food safety after getting pregnant	556	94.40
Willing to learn about food safety knowledge related to pregnancy	538	91.34
Would seek prompt medical care for pregnancy-related symptoms during pregnancy, such as diarrhea or fever	519	88.12
Be willing to undergo necessary LM tests conducted by doctors when seeking medical treatment	431	73.17

#### 3.2.4 High-risk behaviors related to listeriosis among pregnant women

**Figure 6:**
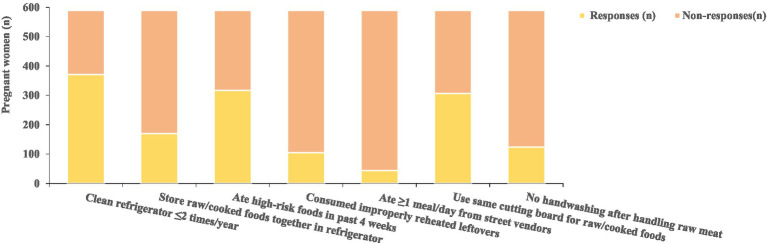
Listeriosis risk behaviors in pregnancy n (%).

Among the surveyed pregnant women, 62.99% of households clean their refrigerators no more than twice per year and 28.86% fail to maintain separation in refrigerators; Additionally, 53.82% reported consuming high-risk LM foods within the 4 weeks prior to the survey, Furthermore, 17.83% have consumed leftover meals from the refrigerator that were not adequately reheated, and 7.47% consume at least one daily meal at roadside or mobile food vendors. In terms of hygiene practices, 52.12% of households do not separate raw and cooked foods on cutting boards, 21.05% of pregnant women or their family members do not wash hands after handling raw meat before proceeding with other cooking steps (see Figure 6 and Table 6).

**Table 6:** Listeriosis risk behaviors in pregnancy n (%).

Risk behavior	Responses (n)	%
Clean refrigerator ≤2 times/year	371	62.99
Store raw/cooked foods together in refrigerator	170	28.86
Ate high-risk foods in past 4 weeks	317	53.82
Consumed improperly reheated leftovers	105	17.83
Ate ≥1 meal/day from street vendors	44	7.47
Use same cutting board for raw/cooked foods	307	52.12
No handwashing after handling raw meat	124	21.05

#### 3.2.5 Comparison of high-risk behaviors among pregnant women across different characteristics

Pregnant women with higher education levels were more likely to engage in high-risk behaviors such as consuming high-risk foods and cleaning refrigerators ≤2 times/year in the 4 weeks before the survey, compared to those with lower education levels. In contrast, lower-educated pregnant women showed higher rates of using the same cutting board for raw and cooked foods and poor handwashing habits. All differences were statistically significant (χ^2^ = 13.920, 7.901, 13.177, 9.939; all *p* < 0.05).

Five high-risk behaviors—including eating at roadside or mobile food stalls, consuming inadequately reheated leftovers, infrequent refrigerator cleaning, not washing hands after handling raw meat, cross-contamination on cutting boards—differed significantly across income groups (χ^2^ = 12.708, 23.402, 9.427, 34.838, 18.765; all *p* < 0.05).

First-time pregnant women were more likely to eat at mobile food stalls, consume high-risk foods, and clean refrigerators≤2 times/year than non-first-time mothers (χ^2^ = 4.267, 10.436, 14.150; all *p* < 0.05). Conversely, non-first-time mothers had a higher rate of consuming underheated leftovers (χ^2^ = 5.094; *p* = 0.036). Pregnant women in the first trimester were more likely to eat at mobile food stalls than those in the second or third trimesters (χ^2^ = 9.925; *p* = 0.031) (see Supplementary Table 1).

#### 3.2.6 Correlation analysis of knowledge, attitude, and behavior related to listeriosis among pregnant women

The average total score for listeriosis related knowledge, attitude, and behavior among pregnant women was 15.52 ± 2.953, with a maximum score of 23 and a minimum score of 7. The median scores (interquartile range) for knowledge, attitude, and behavior were [0 (0–2)], [8 (7–8)], and [7 (5.5–8)], respectively. Spearman correlation analysis revealed a statistically significant positive correlation between knowledge and attitude (*r* = 0.182, *p* < 0.001), as well as a weaker but still significant correlation between knowledge and behavior (*r* = 0.094, *p* = 0.022). However, no significant correlation was observed between attitude and behavior (*r* = −0.038, *p* = 0.053).

#### 3.2.7 Analysis of influencing factors on knowledge, attitude, and behavior regarding listeriosis among pregnant women

A multiple linear stepwise regression analysis was performed to examine the relationship between the overall scores of knowledge, attitude, and behavior regarding listeriosis among pregnant women and selected sociodemographic factors, including age, educational level, family income, gestational age, and parity (whether it was their first pregnancy). The results indicated that all of these factors had a statistically significant influence on the overall score. Specifically, higher scores were associated with older maternal age, higher educational level, greater family income, more advanced gestational age, and being a primiparous woman (first-time mother). The derived linear regression equation is as follows: Y = 8.859 + 0.888X₁ + 0.420X_2_ + 0.319X_3_ + 0.424X_4_ + 0.079X_5_.

## 4 Discussion

China launched a nationwide pilot surveillance program for listeriosis across six provinces /municipalities in 2013. Data from the National Risk Assessment Center (2013–2019) revealed 211 confirmed listeriosis cases across 64 sentinel hospitals in 11 provinces, with notable inter-provincial variation in reported case numbers. Between 2015 and 2020, Henan Province reported 71 listeriosis cases from 16 sentinel hospitals, including 38 perinatal infections (12). Research reports suggest that listeriosis in mainland China is underestimated, with perinatal cases likely being significantly higher than reported (13). Gansu Province incorporated listeriosis into its public health surveillance system in 2022. Since then, a pilot surveillance program has been implemented across five medical institutions in the province. In 2023, Qinyang People’s Hospital identified two pregnancy-associated cases, including one miscarriage and one preterm delivery. Wuwei Liangzhou Hospital reported one pregnancy-associated case involving a live birth at 27 weeks of gestation. The remaining three participating institutions did not report any pregnancy-related cases during the same period. No pregnancy-associated cases of listeriosis were detected in Gansu Province between 2024 and 2025. In certain cases, the reporting of listeriosis is contingent upon the diagnostic vigilance of clinicians. Among 207 surveyed tertiary hospital physicians in current study, only 14.98% had prior experience treating listeriosis, 26.57% were familiar with common LM-contaminated foods, 24.15% could recognize listeriosis symptoms, and 27.54% knew the appropriate treatment drugs. However, medical staff in Gansu Province demonstrated a relatively higher awareness of susceptible populations (46.86%) compared to Beijing’s foodborne disease surveillance personnel (38.78%) in 2016 (14). He et al. identified nonspecific clinical presentations, limited physician experience, and insufficient laboratory diagnostics as key factors contributing to underreporting and misdiagnosis of notifiable infectious diseases (15). Similarly, this study found that missed listeriosis diagnoses primarily resulted from lack of clinical suspicion and inadequate hospital testing capacity. Furthermore, most physicians had not received any listeriosis related training or continuing education in the past 2 years, which may contribute to underdiagnosis and underreporting of listeriosis cases in Gansu Province. Like many foodborne illnesses, Listeriosis often presents with nonspecific symptoms—vomiting, diarrhea, fever, abdominal pain—necessitating thorough clinical and epidemiological evaluation alongside laboratory confirmation (16). Overall, to effectively improve the efficiency of listeriosis surveillance in Gansu Province, it is essential to strengthen training for clinicians on listeriosis, enhance their diagnostic vigilance, and expand the current surveillance network to include more healthcare facilities. Furthermore, The standard treatment for listeriosis in humans involves *β*-lactams, particularly aminopenicillins like ampicillin or amoxicillin, typically combined with an aminoglycoside, most often gentamicin, to enhance the synergistic bactericidal effect (17), Notably, cephalosporins—frequently used as empirical broad-spectrum antibiotics—are ineffective against LM due to intrinsic resistance (8). Therefore, improving the vigilance of clinicians for listeriosis and ensuring adequate empirical antimicrobial coverage for LM is critically important for preventing adverse pregnancy outcomes among affected pregnant women.

This survey revealed that 23.09% of the surveyed pregnant women were aware of or had heard about listeriosis, a rate higher than that reported in Beijing (18), yet significantly lower compared to findings from countries such as the Netherlands (19, 20). The willingness of pregnant women to acquire knowledge about listeriosis and to seek timely medical attention upon experiencing symptoms indicates a positive attitude, which facilitates the implementation of health education initiatives related to listeriosis among pregnant women. LM is capable of growth and reproduction at lower refrigeration temperatures (21), suggesting that refrigeration does not completely prevent food contamination. The results indicated that over 60% of pregnant women cleaned their refrigerators no more than twice per year, particularly among those with higher educational attainment, higher household income, and those who were not primigravid. Additionally, 17.83% of the surveyed pregnant women reported consuming leftover meals from the refrigerator without adequate reheating, predominantly among those with lower household income and primigravid women. Therefore, while promoting comprehensive listeriosis related health education for all pregnant women, targeted interventions should be emphasized for these specific subgroups. Efforts should focus on encouraging regular refrigerator cleaning and maintaining hygiene to prevent prolonged neglect, which may lead to LM proliferation and subsequent contamination of food, thereby posing potential risks to human health.

Machine-made ice cream, Chinese cold dishes, and ready-to-eat cooked meat products are recognized as high-risk food items for LM contamination (22). According to national surveillance data from 2021, the detection rate of LM in ready-to-eat cooked meat products across China was 2.49% (23). In Changchun City, between 2010 and 2023, the reported detection rate of LM in commercially available cold dishes reached 9.49% (24). This study found that more than half of the participants had consumed these high-risk foods within the past 4 weeks, with a higher proportion among those with higher educational attainment and first-time pregnant women. Given the relatively low incidence of listeriosis and its non-specific clinical presentation upon infection, public awareness of LM remains limited. Therefore, implementing targeted health education initiatives focusing on pregnant women is of critical importance.

Listeriosis is a preventable foodborne illness that can be mitigated through standard food safety practices, such as separating raw and cooked foods and ensuring thorough heating of food before consumption. In this survey, over half of the participating pregnant women did not maintain separate cutting boards for raw and cooked ingredients, and some failed to practice proper hand hygiene after handling raw meat during food preparation. Given that raw meat and processed meat products are recognized as high-risk vehicles for LM contamination, adherence to basic food safety principles is essential. A systematic review conducted by Zhang et al. revealed that the prevalence of LM in livestock and poultry meat in northern China was consistently higher than in southern regions (25). The positive detection rates of LM in ice cream and Chinese cold mixed dishes in Gansu Province from 2007 to 2011 were 8.33 and 3.14%, respectively (26). In 2022, the detection rate of LM in processed meat products—such as chicken and duck meat links, chicken fillets, and restructured beef steaks—was 9.16%, while raw poultry meat showed a detection rate of 12.11%, indicated raw meat and processed meat products may be susceptible to contamination by LM. Inadequate hygiene practices, such as improper separation of raw and cooked foods or failure to wash hands after handling raw meat, may lead to cross-contamination of cooked or ready-to-eat foods, thereby increasing the risk of human infection. Therefore, cultivating good food hygiene habits is critical for the prevention of listeriosis and other foodborne diseases. Moreover, the study found that a relatively small but notable proportion of pregnant women consume at least one daily meal from roadside stalls or mobile food vendors. While such food sources offer convenience and accessibility, they often lack adequate food safety controls. As a vulnerable population group, the nutritional and health status of pregnant women significantly influences both maternal and fetal well-being. Therefore, targeted interventions to guide pregnant women in making scientifically informed food choices and selecting appropriate dining venues are essential for preventing foodborne disease occurrence.

The Knowledge-Attitude-Practice (KAP) theory describes a sequential process in which individuals acquire knowledge, develop attitudes or beliefs, and ultimately form corresponding behaviors, with a positive interrelationship among these components. This study revealed a significant positive correlation between knowledge and attitude regarding listeriosis among pregnant women; however, no significant association was observed between attitude and behavior. These findings suggest that pregnant women in Gansu Province demonstrate coherence in acquiring listeriosis related knowledge and forming preventive beliefs. Although they tend to develop favorable attitudes toward listeriosis prevention after gaining relevant knowledge, this attitudinal shift does not appear to translate into consistent preventive actions. This discrepancy may stem from a mismatch between the knowledge acquired and the specific preventive measures required for listeriosis, or from an inadequate understanding of high-risk behaviors associated with the disease. Therefore, future health education initiatives on listeriosis should emphasize clear communication of actionable preventive strategies to enhance recipients’ comprehension and promote behavior change. Additionally, our survey found that older pregnant women with higher education levels, better family incomes, and those who are further along in their pregnancies—or experiencing their first pregnancy—tend to have better knowledge, attitudes, and behaviors when it comes to listeriosis. That means while we should keep providing health education to all pregnant women, we should especially focus on younger moms-to-be, those with less education, lower income, those in their early pregnancy, and those who have been pregnant before. Guiding the population to establish good hygiene habits and abandon high-risk behaviors of listeriosis is of great significance for avoiding adverse pregnancy outcomes.

Ongoing attention will be given to the surveillance and reporting of listeriosis cases in Gansu Province.

## Data Availability

The original contributions presented in the study are included in the article/Supplementary material, further inquiries can be directed to the corresponding author.
