# Supplementary material for: A cross-sectional study on clinical vigilance in the diagnosis and treatment of listeriosis among pregnant women and their knowledge, attitudes, and practices regarding listeriosis in Gansu Province, China
Source: Front Public Health. 2026 Jan 8;13:1661344. doi: 10.3389/fpubh.2025.1661344 (PMC12823871; doi:10.3389/fpubh.2025.1661344)
Supplement: Supplementary file 1 [file Table_1.pdf]

TABLE 7 Sociodemographic variations in Listeriosis risk behaviors among pregnant women n (%)

**Characteristics**	**Clean refrigerator ≤2 times/year** n (%)	**Ate high-risk foods in past 4 weeks** n (%)	**Use same cutting board for raw/cooked foods** n (%)	**Store raw/cooked foods together in refrigerator** n (%)	**No handwashing after handling raw meat** n (%)	**Consumed improperly reheated leftovers** n (%)	**Ate ≥1 meal/day from street vendors** n (%)
**Age (years)**							
18-29	184（63.67）	167（57.79）	141（48.79）	89（30.80）	65（22.49）	51（17.65）	26（9.00）
30-39	181（61.99）	148（50.68）	163（55.82）	80（27.04）	57（19.52）	53（18.15）	18（6.16）
≥40	6（75.00）	2（25.00）	2（25.00）	1（12.50）	2（25.00）	1（12.50）	0（0.00）
χ^2	0.585	0.539	5.127	1.597	1.052	0.081	1.681
P	0.777	0.06	0.073	0.462	0.617	0.969	0.348
**Education level**							
College/University	265（65.76）	234（58.06）	189（46.90）	110（27.30）	73（18.11）	63（15.63）	29（7.20）
High School/Vocational	75（61.98）	61（50.41）	75（61.98）	44（36.36）	38（31.40）	27（22.31）	10（8.26）
Middle School or below	31（47.69）	22（33.85）	42（64.62）	16（24.62）	13（20）	15（23.08）	5（7.69）
χ^2	7.901	13.920	13.177	4.369	9.939	4.211	0.293
P	**0.019**	**0.001**	**0.001**	0.115	**0.007**	0.122	0.856
**Monthly household income (¥)**							
< 2000	24（60.00）	23（57.50）	25（62.50）	17（42.50）	23（57.50）	18（45.00）	2（5.00）

2,000-4,999	88（54.32）	74（45.68）	103（63.58）	52（32.10）	33（20.37）	29（17.90）	9（5.56）
5,000-9,999	203（68.58）	169（57.09）	143（48.31）	73（24.66）	52（17.57）	41（13.85）	17（5.74）
≥10,000	56（61.54）	51（56.04）	35（38.46）	28（30.77）	16（17.58）	17（18.68）	16（17.58）
χ^2	9.427	5.996	18.765	7.154	34.838	23.402	12.708
P	**0.024**	0.112	**0.000**	0.067	**0.000**	**0.000**	**0.004**
**Pregnancy history**							
Pregnant for the first time	211（70.33）	181（60.33）	144（48.00）	88（29.33）	55（18.33）	43（14.33）	29（9.67）
Pregnant before	160（55.36）	136（47.06）	162（56.06）	82（28.37）	69（23.88）	62（21.45）	15（5.19）
χ^2	14.150	10.436	3.826	0.066	2.720	5.094	4.267
P	**0.000**	**0.001**	0.058	0.856	0.099	**0.031**	**0.039**
**Gestational age (weeks)**							
<12	36（59.02）	31（50.82）	33（54.10）	18（29.51）	11（18.03）	13（21.31）	10（16.39）
12-27	150（66.08）	118（51.98）	114（50.22）	68（29.96）	50（22.03）	39（17.18）	16（7.05）
≥28	185（61.46）	168（55.81）	159（52.82）	84（27.91）	63（20.93）	53（17.61）	18（5.98）
χ^2	1.644	1.011	0.477	0.278	0.467	0.580	6.925
P	0.450	0.603	0.792	0.872	0.807	0.764	**0.031**
